# Perinatal Exposure to Tobacco Smoke and Its Association with the Maternal and Offspring Microbiome: A Systematic Review

**DOI:** 10.3390/healthcare12181874

**Published:** 2024-09-19

**Authors:** Eleni Falara, Dimitra Metallinou, Christina Nanou, Maria Vlachou, Athina Diamanti

**Affiliations:** Department of Midwifery, Faculty of Health and Caring Sciences, University of West Attica, 12243 Egaleo, Greece; aebmc18041@uniwa.gr (E.F.); dmetallinou@uniwa.gr (D.M.); nanouxv@uniwa.gr (C.N.); adiamanti@uniwa.gr (A.D.)

**Keywords:** smoking, tobacco smoke, pregnancy, microbiome, microbiota, perinatal period, offspring

## Abstract

Background: The human microbiome, comprising trillions of microorganisms, significantly influences human health and disease. During critical periods like the perinatal phase, the microbiome undergoes significant changes, impacting lifelong health. Tobacco smoke, a known environmental pollutant, has adverse effects on health, particularly during pregnancy. Despite this, its association with the perinatal microbiome remains understudied. Methods: We conducted a systematic review to integrate findings on perinatal tobacco smoke exposure and its association with the maternal and neonatal microbiomes. We conducted a comprehensive literature search in the PubMed, Scopus, and Web of Science databases from January 2000 to February 2024. We selected studies that met predefined inclusion criteria and performed data extraction. Results: The review included eight studies that revealed diverse associations of perinatal tobacco exposure with the maternal and neonatal microbiome. Active smoking during pregnancy was linked to alterations in microbiome composition and diversity in children. Maternal smoking correlated with increased Firmicutes abundance and decreased *Akkermansia muciniphila* abundance in offspring. Additionally, exposure to thirdhand smoke in neonatal intensive care units was related to infant microbiome diversity. Infants exposed to tobacco smoke showed various microbial changes, suggesting potential implications for childhood health outcomes, including obesity risk. Conclusions: Perinatal exposure to tobacco smoke exerts significant influence on the maternal and neonatal microbiomes, with potential implications for long-term health outcomes. Addressing socioeconomic and psychological barriers to smoking cessation, implementing stricter smoking regulations, and promoting public health campaigns are essential steps towards reducing tobacco-related harm during the perinatal period. Further longitudinal studies and standardized assessment methods are needed to validate these findings and guide the development of effective preventive measures.

## 1. Introduction

The human microbiome, consisting of trillions of microorganisms that inhabit our bodies, is an essential factor in human health and disease. These microorganisms, which include bacteria, viruses, fungi, and other microbial entities, are crucial in regulating various bodily functions such as immune response, digestion, and the synthesis of vital nutrients [1,2]. The composition and diversity of the microbiome can influence an individual’s susceptibility to a range of conditions, from autoimmune diseases to metabolic syndromes [3,4].

During critical developmental periods, such as the perinatal phase—which encompasses the time before and shortly after birth—the microbiome undergoes significant changes. In pregnant women, the maternal microbiome not only supports maternal health but also plays a decisive role in fetal development and the establishment of the neonatal microbiome [5,6,7]. Researchers believe that the early microbiological environment shapes the child’s lifelong health trajectory by influencing immune system maturation, metabolic programming, and even behavioral development [8,9].

Tobacco smoke is a complex mixture of thousands of compounds, many of which are toxic and have been shown to adversely affect human health. Previous research well documents its role as a major environmental pollutant and risk factor for numerous diseases, including respiratory conditions, cardiovascular diseases, and various forms of cancer [10,11,12]. Furthermore, studies have linked exposure to tobacco smoke during pregnancy to several adverse perinatal outcomes, including preterm birth, low birth weight, and developmental delays [13,14]. However, the impact of tobacco smoke on the microbiome during the perinatal period remains a relatively under-explored area of research.

Given the established critical roles of both the microbiome and environmental tobacco smoke in health and disease, it is imperative to understand how perinatal exposure to tobacco smoke influences the microbial communities in the mother and her child. This understanding could reveal new mechanisms by which tobacco exposure affects offspring’s developmental and health outcomes. Additionally, it could potentially lead to interventions that mitigate these adverse effects through microbiome modulation.

This research aims to systematically review the available scientific literature to integrate findings related to perinatal exposure to tobacco smoke and its association with the maternal and neonatal microbiome. By consolidating current knowledge and identifying gaps in the literature, this review seeks to highlight the complex interactions between environmental toxins and microbial ecology during one of the most crucial stages of human development.

## 2. Materials and Methods

### 2.1. Search Strategy

To identify relevant studies examining association of perinatal exposure to tobacco smoke with the maternal and child microbiome, a comprehensive literature search was conducted using the PubMed, Scopus, and Web of Science databases according to the Preferred Reporting Items for Systematic Reviews and Meta-Analyses (PRISMA) [15]. The search included peer-reviewed articles published in English from January 2000 to February 2024. The Medical Subject Headings (MeSH) terms used were a combination of: (“perinatal exposure” OR “maternal smoking” OR “tobacco smoke exposure”) AND (“microbiome” OR “microbial” OR “gut microbiota”) AND (“infant” OR “child” OR “neonatal” OR “maternal”). References to selected articles were also manually searched to identify additional studies. The screening of the records was conducted based on title, abstract, and whether the full text met all the predetermined criteria as per the PICOs: P (Population): Pregnant women and their neonates, I (Intervention): Perinatal tobacco smoke exposure (active maternal smoking, secondhand smoke exposure, thirdhand smoke exposure in NICU), C (Comparison): Pregnant women and neonates with no exposure to tobacco smoke, O (Outcomes): Composition and diversity of the maternal and neonatal microbiome (including specific bacterial taxa like Firmicutes and *Akkermansia muciniphila*), and Potential long-term health outcomes associated with microbiome changes (e.g., obesity risk). This systematic review was prospectively registered with PROSPERO. Registration number: CRD42024558857.

### 2.2. Eligibility Criteria

Studies were included if they: (a) were original research articles; (b) studied the association with perinatal tobacco smoke exposure; (c) examined the microbiome of either the mother or the child; (d) used direct microbial profiling methods such as 16S rRNA gene sequencing or metagenomic sequencing; (e) were published in the English language; and (f) had full-text availability.

Studies were excluded if they: (a) were review articles, opinions, or editorials; or (b) did not provide quantitative data on microbiome changes.

### 2.3. Selection of Studies

Two reviewers autonomously evaluated the titles and abstracts of the retrieved articles using the search criteria. Any discrepancies between the reviewers were resolved through discussion, and if a consensus could not be reached, a third assessor was consulted to make the final decision. The full texts of potentially suitable studies were obtained and scrutinized for inclusion based on predefined criteria.

### 2.4. Evaluation of Validity

All articles underwent assessment using the Strengthening the Reporting of Observational Studies in Epidemiology (STROBE) criteria [16] designed for systematic reviews. We reviewed and evaluated aspects such as methodology appropriateness, the study design, data collection, potential biases, clarity, and the validity of the findings. The results of this evaluation are presented in Supplementary Table S1. 

### 2.5. Data Extraction

From the included studies, the following data were extracted: (a) first author’s name; (b) year of publication; (c) study design (prospective, cross-sectional, case-control, etc.); (d) sample size; (e) biological sample examined; (f) the microbial profiling method used; and (g) key findings related to the impact of tobacco exposure on the microbiome.

## 3. Results

**Figure 1:**
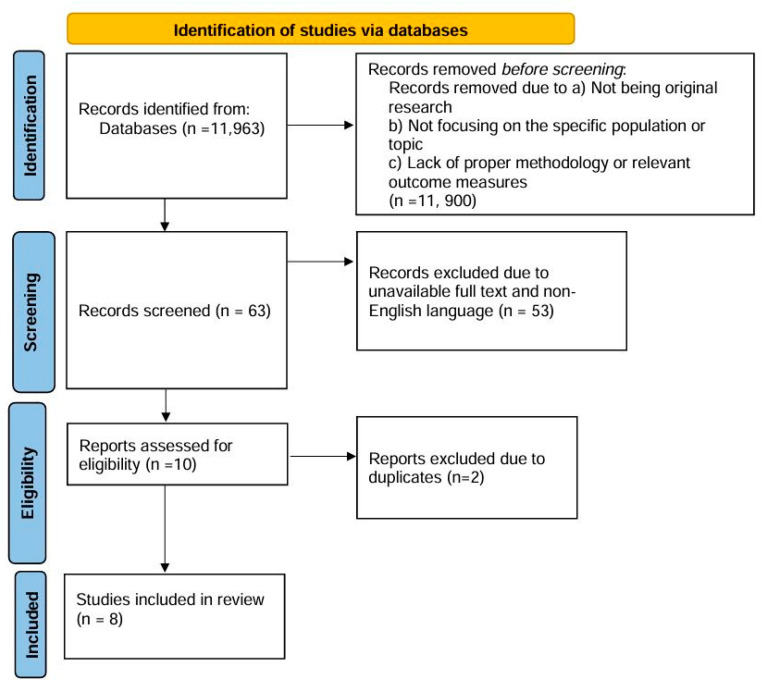
Flow chart detailing the methodological approach, including the search strategy and the screening process.

Initially, a total of 11,963 records were identified through comprehensive database searches. Before any screening was conducted, 11,900 records were excluded due to reasons not specified in the eligibility criteria, such as irrelevance to the topic of study. Of the remaining records, 63 were screened for further evaluation. At this stage, 53 records were excluded due to being either non-English or lacking full-text availability, leaving only 10 records to be assessed in detail. Upon detailed assessment, two records were found to be duplicates and were removed from consideration. This resulted in eight records being eligible for inclusion. The final selection included eight studies that met all the inclusion criteria. A flow chart detailing the methodological approach, including the search strategy and the screening process, is depicted in Figure 1.

The comprehensive review incorporated studies that examined the association of perinatal exposure to tobacco smoke with the microbiome of mothers and their children. These studies utilized various microbial profiling methods to assess changes in the microbiome due to tobacco exposure. A summary of the study characteristics and findings is presented in Table 1.

Table 2 summarizes specific characteristics of the population evaluated in the included studies.

Perez-Castro et al. in their exposome-wide study investigated the persistent influence of prenatal tobacco and mercury exposure on the fecal microbiome composition of 7-year-old children, considering various lifestyle and clinical factors. Gut microbiome analysis was conducted in 151 children using 16S rRNA sequencing at the genus level. Results revealed that active smoking during pregnancy was consistently associated with microbiome composition and diversity, independent of other factors. However, no significant associations were found for mercury exposure or tobacco exposure during childhood. Notably, smoking during pregnancy correlated with increased Dorea abundance and decreased *Akkermansia* abundance. These findings suggest a lasting relationship between prenatal tobacco exposure and children’s gut microbiota, highlighting the need for further investigation into the role of environmental exposures on microbiota composition [17].

Gosalbes et al. aimed to characterize the meconium microbiota in term infants using molecular techniques, assess its contribution to future gut colonization, and evaluate its relationship with maternal and infant health factors. High-throughput sequencing of the 16S rRNA gene was conducted on meconium samples from 20 infants, revealing two distinct types of microbiota. One type was dominated by enteric bacteria and associated with maternal history of atopic eczema, while the other was dominated by lactic acid bacteria and associated with infant respiratory problems. These findings suggest that the meconium microbiota has an intrauterine origin, influenced by maternal factors, and may have implications for childhood health [18].

Northrup et al. examined the impact of Thirdhand Smoke (THS) exposure on the gut microbiomes of infants admitted to a Neonatal Intensive Care Unit (NICU). Forty-three mother–infant pairs participated, with varying levels of household smoking. The results revealed that infants from households where smoking was not present or NICU furniture nicotine levels were lower, exhibited higher microbiome diversity compared to those from smoking households. Additionally, associations were observed between various bacterial genera and urine cotinine levels, surface nicotine levels, and household smoking. Notably, lower Bifidobacterium abundance was linked to higher furniture nicotine and household smoking. These findings suggest that THS exposure is related to NICU infants’ gut microbiomes, emphasizing the need for further research on the association of tobacco with healthy infant microbiome development [19].

Huotari et al. examined a cohort of newborn infants, with 3.8% of mothers reporting smoking during pregnancy. Infants exposed to maternal smoking exhibited higher relative abundances of Bacteroidetes and Proteobacteria phyla, along with lower abundance of Firmicutes. Furthermore, their gut microbiome showed lower diversity and fewer operational taxonomic units (OTUs). Based on this and existing literature, we propose further evaluation of the association between maternal smoking and early gut microbiome in newborns, as it may have clinically significant implications [20].

The study by Tun et al. examined the impact of tobacco smoke exposure on the gut microbiota of 959 infants. They found increased species richness of Firmicutes in infants exposed to smoke postnatally or both pre- and postnatally. Their analysis suggested that changes in the microbiota might mediate the relationship between smoke exposure and the risk of childhood overweight at ages 1 and 3, indicating a significant association of environmental tobacco smoke with the gut microbiome and child health outcomes [21].

The study by Xie et al. [22] examined the effects of environmental tobacco smoke (ETS) exposure and breastfeeding duration on infant gut microbiota in China. Over 2 years, 37 mother–child pairs were followed, with data collected on ETS exposure, breastfeeding duration, and fecal samples analyzed via 16S rRNA sequencing. 

The findings indicated that extended breastfeeding correlated with elevated levels of advantageous microbes like Lactobacillus and reduced levels of detrimental bacteria such as Clostridium sensu stricto 1. Moreover, children who were not exposed to smoke exhibited greater diversity in their microbiota and higher concentrations of beneficial bacteria.

Levine et al. explored the joint impact of various factors on the gut microbiome of infants from a diverse Detroit-based birth cohort. The gut microbiota of 298 children underwent analysis, uncovering correlations with indoor pets, maternal race–ethnicity, mode of delivery, breastfeeding, marital status, and exposure to environmental tobacco smoke. These factors independently influenced microbiome composition in both neonates and infants, with combinatorial effects further explaining variation. The study underscores the significance of considering multiple exposures in understanding early-life gut microbiota and its potential implications for disease studies [23].

Peng et al. examined the association between maternal smoking during pregnancy, the composition of gut microbiota, and the risk of childhood obesity. Their analysis of data from 1592 infants indicated that discontinuing smoking during pregnancy did not decrease the likelihood of childhood overweight. However, exclusively breastfeeding until the third month of age was linked to reduced risks. Maternal smoking during pregnancy was found to elevate both the abundance and diversity of Firmicutes in the gut microbiota. Notably, the diversity of Firmicutes was identified as a mediator for the heightened risk of childhood overweight and obesity [24].

## 4. Discussion

Our systematic review showed a complex relationship between maternal smoking and significant changes in the infant microbiota. An increased proliferation of Firmicutes and a marked decrease in beneficial microbes, particularly *Akkermansia muciniphila*, characterize these alterations. The role of *Akkermansia* is pivotal, as it is known to enhance mucosal function and support metabolic processes essential for maintaining gastrointestinal integrity and preventing obesity [25]. Conversely, the increased presence of Firmicutes correlates with enhanced caloric extraction from the diet, potentially predisposing individuals to obesity in later life [26]. This evidence suggests that exposure to tobacco smoke during critical developmental windows may imprint lasting association with the microbiome, potentially setting a trajectory towards increased disease risk in adulthood.

The pathways through which tobacco exposure influences microbiome development are diverse and complex. Tobacco smoke contains a myriad of chemical constituents capable of directly altering the gut environment or indirectly affecting it through modifications in local immune responses [27]. For instance, nicotine and other smoke components can escalate oxidative stress within the gut, fostering an environment conducive to the proliferation of pathogenic bacteria while inhibiting commensal species [28]. Additionally, the impact of smoking on maternal immune function may significantly affect the patterns of microbial transmission and colonization in the infant gut, potentially disrupting normal immune system development and microbiota maturation [29].

The well-documented negative impact of nicotine on newborn body weight is widely acknowledged. This influence is primarily attributed to nicotine’s vasoconstrictive effects on the placenta, leading to fetal hypoxia. Additionally, nicotine can traverse the placenta, affecting fetal immune balance. Evidence of nicotine compounds in the meconium of neonates born to mothers who reported tobacco use further underscores its prenatal exposure [30,31,32,33]. In adults, cigarette use or exposure fosters the proliferation of certain bacterial genera in the gut, such as Bacteroides, Prevotella, Enterobacteria, and Clostridium [34]. Interestingly, changes in gut microbiota composition, specifically an increase in Firmicutes and a decrease in Bacteroidetes, have been associated with weight gain post-smoking cessation in adults, mirroring obesity patterns observed in animal studies [34]. Remarkably, tobacco curing for cigarette production involves microbes that contribute to flavor and fragrance biosynthesis, with recent gene sequencing studies identifying various Bacilli species on cured tobacco [35]. Given these insights into the gut microbiota composition of smokers, it is plausible that observed alterations are influenced by microbes present in cigarette tobacco, either directly or indirectly, through microbial competition.

Childhood obesity frequently arises from dysregulation in early-life gut microbiota. Research indicates that disturbances in microbiota composition as early as 1 month postnatally may heighten the likelihood of developing chronic illnesses later in life. While research on infant gut microbiota and child overweight is limited, research has demonstrated changes in the gut microbiota of infants who later develop overweight [36]. Cigarette smoking, a significant modifiable risk factor linked to pregnancy complications and child overweight, warrants attention [37]. Identifying environmental factors like maternal smoking, which can alter an infant’s microbial composition, presents an opportunity to mitigate the risk of child overweight and obesity.

Building upon the identified interactions between perinatal tobacco exposure and microbiome development necessitates a comprehensive approach to public health interventions. Integrating smoking cessation programs into prenatal care could substantially decrease the prevalence of maternal smoking. These programs are not only cost-effective but also beneficial for both maternal and neonatal health outcomes [38].

Research from Higgins et al. [39] underscores the importance of addressing socioeconomic and psychological barriers in smoking cessation programs. These barriers often impede quitting efforts, suggesting that personalized interventions might be more effective. Additionally, the inclusion of psychological support and counseling in these programs can significantly enhance cessation rates [40], thereby minimizing the exposure of both mother and child to harmful tobacco smoke.

The long-term health ramifications of microbiome alterations caused by maternal smoking warrant more attention than immediate health outcomes. Research by Vrijheid et al. [41] has linked early microbiome changes to the later development of conditions such as asthma and allergies, highlighting the necessity for early intervention strategies. Moreover, the findings by Logan et al. [42] suggest that the repercussions of maternal smoking may extend beyond physical health, impacting cognitive and neurological development.

Improving laws concerning smoking in public areas and near children can greatly reduce the exposure to passive smoke. Been et al. [43] documented the efficacy of such regulatory measures in reducing pediatric health issues, observing a decline in asthma exacerbations following the enforcement of stricter smoking bans. Public health campaigns that effectively communicate the risks associated with prenatal and postnatal tobacco exposure could also shift societal norms and behaviors regarding smoking among women and children.

Future research should concentrate on elucidating the precise biological mechanisms through which tobacco exposure affects the infant microbiome and identifying optimal windows for intervention during gestation and early life. Understanding critical periods of microbiome vulnerability could lead to targeted therapies aimed at maintaining or restoring microbiome health [44]. Furthermore, longitudinal studies are needed to confirm these findings and delve deeper into the mechanisms driving microbiome changes [45].

Despite the robust findings, several limitations within the reviewed studies warrant consideration. Firstly, the reliance on self-reported data for smoking exposure introduces potential recall and reporting biases. Despite their use in some studies, the uniform application of objective measures like cotinine levels could potentially affect the consistency of exposure assessment across the studies. Secondly, the studies’ designs, predominantly observational, limit causal inferences. Although longitudinal studies provide strong evidence for temporal associations, they cannot definitively establish causality because of potential residual confounding of data from unmeasured variables such as diet, socioeconomic status, and other lifestyle factors.

Another critical limitation is the variation in sample collection timing across studies, which could affect the comparability of results. The microbiome is known to develop dynamically over the first years of life; thus, variations in the timing of assessments can lead to different conclusions regarding the impact of tobacco exposure. In this systematic review, 53 potentially relevant papers were excluded due to unavailability or language barriers. This represents a significant limitation and potential source of bias. The exclusion of these studies may have restricted the diversity of the data analyzed, as non-English language studies or those not easily accessible could present findings that differ from the studies included in the review. As a result, the generalizability of our findings might be affected. Future systematic reviews should aim to incorporate a more comprehensive set of studies by using translation services or ensuring broader database access to minimize this limitation.

## 5. Conclusions

In conclusion, the interplay between maternal smoking during pregnancy and the neonatal microbiome presents a complex yet critical area of study with far-reaching implications for human health. Our systematic review revealed significant alterations in the gut microbiota of infants exposed to tobacco smoke, characterized by increased Firmicutes abundance and decreased levels of beneficial microbes like *Akkermansia muciniphila*. In later life, these changes may set a trajectory towards increased disease risk, including childhood overweight and obesity. Understanding the underlying mechanisms driving these microbiome alterations is essential for developing targeted interventions aimed at mitigating adverse health outcomes associated with tobacco exposure. The pathways through which tobacco smoke influences microbiome development are multifaceted, involving direct alterations in the gut environment and modifications in local immune responses. Moreover, the long-term health ramifications of these microbiome alterations underscore the urgency of implementing effective public health interventions targeting smoking cessation and reducing second and thirdhand smoke exposure, particularly during pregnancy and early childhood.

## Figures and Tables

**Table 1:** Detailed summary of studies on perinatal tobacco exposure and microbiome changes.

*n*	First Author, Year	Study Design	Establishment of Tobacco Exposure	Sample Size	Biological Sample	Statistical Method	Microbial Profiling Method	Microbiome Changes Observed
1	Pérez-Castro et al., 2024 [17]	Prospective	Exposure to tobacco was quantified during pregnancy through questionnaire (active tobacco consumption, secondhand smoking -SHS) and biomonitoring (urinary cotinine) at 4 years (urinary cotinine, SHS) and 7 years (SHS).	151 mother–child pairs	Children’s fecal samples at 7 years of life	Statistical methods include PERMANOVA for diversity measures and MaAsLin2 for genus-level associations, adjusted for covariates like maternal education and BMI.	16S rRNA gene sequencing	Active smoking in pregnancy was systematically associated with microbiome composition and ß-diversity. More specifically, active smoking at any time during pregnancy was associated with a decrease in the relative abundance of *Akkermansia muciniphila*. Sustained maternal smoking from the first to the third trimester of pregnancy was associated with an increased relative abundance of Dorea.
2	Gosalbes et al., 2013 [18]	Prospective	Maternal questionnaire: Smoked during early pregnancy (Yes/No) or smoked entire pregnancy (Yes/No)	20 term newborns 7 pregnant women	Meconium microbiota, maternal fecal microbiota	Logistic regression and Mann–Whitney tests for comparing clinical associations with microbiota	16S rRNA gene sequencing	Type A microbiota, which presents the family Enterobacteriaceae as the most abundant bacterial taxon (58.69%), mainly represented by the Escherichia/Shigella genus (24.68%), was the only type of microbiota found in maternal fecal samples of pregnant smokers. Presence of Enterobacteriaceae in meconium microbiota was linked to maternal atopic conditions.
3	Northrup et al., 2022 [19]	Cross-sectional	Participants (*n* = 43) consented to an interview (assessing infant feeding, visitation, and holding, and household cigarette/tobacco/nicotine use and exposure), a carbon monoxide (CO)-breath sample, one bedside (NICU) furniture surface-nicotine wipe, one infant-urine collection (for cotinine [primary metabolite of nicotine] assays)	43 (32 smoking households, 11 non-smoking households)	Stool collection during hospitalization	Use of negative binomial regression, Mann-Whitney U-tests, and Bayesian analyses to model the associations between THS exposure and microbiome diversity.	16S rRNA gene sequencing	Infants from non-smoking homes and/or with lower NICU-furniture surface nicotine had greater microbiome alpha-diversity compared to infants from smoking households. Associations of selected bacterial genera with urine cotinine, surface nicotine, and/or household cigarette use were evidenced for 7 (of 8) modelled genera. For example, lower Bifidobacterium relative abundance associated with greater furniture nicotine.
4	Huotary et al., 2019 [20]	Cross-sectional	Maternal smoking status was verified at one year of age with a separate follow-up questionnaire	131 infants	First-pass meconium microbiome	The Mann-Whitney U-test was used to compare microbial abundances between groups	16S rRNA gene sequencing	The relative abundances of Bacteroidetes and Proteobacteria phyla were higher and the relative abundance of Firmicutes phylum was lower in those infants exposed to maternal smoking during pregnancy. In addition, the infants exposed to maternal smoking had less diverse gut microbiome and the number of the operational taxonomic units (OTUs) was lower.
5	Tun et al., 2017 [21]	Prospective	No exposure; exposure only during pregnancy; exposure only postnatally; and exposure during pregnancy and postnatally	959 infants	Fecal microbiota stool samples at 3–4 month	Statistical analyses included logistic regression for overweight/obesity risk, adjusted odds ratios (OR), and mediation analysis to explore gut microbiota’s role in the association between smoking exposure and obesity.	16S rRNA Illumina MiSeq	↑ Species richness of Fir micutes (Ruminococcaceae and Lachnospiraceae) at 3 months in infants exposed to tobacco smoke postnatally or both pre- and postnatally. Firmicutes richness potentially mediated postnatal smoking exposure and risk of childhood overweight at age 1 and 3 years
6	Xie et al., 2021 [22]	Prospective	Mothers completed general questionnaires pertaining to themselves during their third trimester, after delivery, and at 12 months, and completed questionnaires pertaining to their children at 6, 12, and 24 months after delivery. Smokers were excluded.	37 mother-child pairs	Fecal samples were collected from mothers in their third trimesters and from children at 6 months, 12 months, and 24 months of age.	Statistical analyses included Welch’s *t*-tests to compare alpha diversity between groups, PERMANOVA for beta diversity differences, and multivariate regression models adjusted for confounders.	16S rRNA gene sequencing	The a diversity of microbiota and the relative abundance of [Ruminococcus]_gnavus_group was higher in the non-smoke exposed group. The higher the smoke exposure, the higher the relative abundance of Megasphaera. Prolonged breastfeeding and reduced smoke exposure are beneficial to the diversity and composition of gut microbiota in young children.
7	Levin et al., 2016 [23]	Prospective	Environment exposure to smoke	298 children (168 infants and 130 neonates)	Stool specimens gathered during the first year of life.	Statistical methods included PERMANOVA and zero-inflated negative binomial regression for analyzing the effects of factors on microbiome composition.	16S rRNA gene sequencing	Infants of mothers who smoked either during pregnancy or currently had higher abundances of Bacteroides and Staphylococcus taxa. Neonates currently exposed to (ETS): ↑ Ruminococcus and Akkermansia.
8	Peng et al., 2024 [24]	Prospective	Self-reported data on smoking prior to and/or during pregnancy Concentrations of infant urinary nicotine metabolites, such as cotinine and trans-3′- hydroxycotinine, measured at the age of 3 months	1592 mother- child pairs	Fecal microbiota on 3 months and 1 year	Generalized linear models, mediation analysis, and correlation tests (Pearson’s, Spearman’s) were used to assess the relationship between maternal smoking and obesity.	16S rRNA gene sequencing	The researchers reported that maternal smoking during pregnancy significantly increased Firmicutes abundance and diversity. They further revealed that Firmicutes diversity mediates the elevated risk of childhood overweight and obesity linked to maternal prenatal smoking.

**Table 2:** Specific characteristics of the population evaluated in the included studies.

Study	Age	Gender	Ethnicity	Location
Gosalbes et al. [18]	28–30 (mothers)	Male and Female (infants)	Spanish (mothers)	Valencia, Spain
Huotari et al. [20]	Newborn infants (first stool collected after birth)	Not specified directly in the document	Not specified explicitly	Jyväskylä, Central Finland
Levin et al. [23]	Neonates (1 month, range: 0.5–4.6 months), Infants (6 months, range: 5.6–10.6 months)	Not specified directly	Primarily African American (53%), Caucasian/Other (47%)	Wayne and Oakland counties, Michigan, USA
Northrup et al. [19]	Mothers: Mean age ~30	Female (mothers), Both Male and Female (infants)	Majority African American (53.1%), Hispanic (31.3%), White (9.4%), Other (6.3%)	Houston, Texas, USA
Peng et al. [24]	Infants aged 3.5 ± 1.0 months (early infancy) and 12.3 ± 1.4 months (late infancy)	Both Male and Female	Majority Caucasian (75.8%), Asian (14.8%), Other (9.4%)	Canada
Pérez-Castro et al. [17]	7 years old	Both Male and Female	White European (majority) Others (mixed ethnicities)	Sabadell, Catalonia, Spain
Xie et al. [22]	0–2 years old (infants at different time points: 6, 12, and 24 months)	Both Male and Female	Chinese	Wuhan, Hubei Province, China
Tun et al. [21]	Ιnfants at 3–4 months, overweight assessed at 1 and 3 years	Both Male and Female	Mixed, no specific details provided	Edmonton, Vancouver, Winnipeg, Canada

## Data Availability

The data presented in this study are available within the article.

## Supplementary Materials

The following supporting information can be downloaded at: https://www.mdpi.com/article/10.3390/healthcare12181874/s1, Table S1: Strengthening the Reporting of Observational Studies in Epidemiology (STROBE) criteria for the included studies.
