# Supplementary material for: Perinatal Exposure to Tobacco Smoke and Its Association with the Maternal and Offspring Microbiome: A Systematic Review
Source: Healthcare (Basel). 2024 Sep 19;12(18):1874. doi: 10.3390/healthcare12181874 (PMC11431162; doi:10.3390/healthcare12181874)
Supplement: Supplementary file 1 [file healthcare-12-01874-s001.zip › healthcare-3108539-supplementary.pdf]

Table S1: Strengthening the Reporting of Observational Studies in Epidemiology (STROBE) criteria for the included studies.

Key STROBE Checklist Items	Questions	Gosalbes et al. [18]	Huotari et al. [20]	Levin et al. [23]	Northrup et al. [19]	Peng et al. [24]	Pérez-Castro et al. [17]	Xie et al. [22]	Tun et al. [21]
Title	Does the title clearly indicate the study is observational?	The title clearly reflects the nature of the study, which is an observationa l investigatio n into meconium microbiota and its relation to maternal and infant health	The title reflects the observational nature of the study, investigating the impact of maternal smoking on the infant gut microbiome.	The title reflects the observational nature of the study, investigating multiple factors that impact gut microbiome composition in neonates and infants.	The title reflects the observationa l nature of the study, examining the association of thirdhand smoke exposure with gut microbiome differences in NICU infants.	The title clearly reflects the observational nature of the study, specifically the effects of maternal smoking on gut microbiome-associated childhood obesity.	The title reflects the observational nature of the study, investigating perinatal and childhood exposure to tobacco and mercury and its effect on gut microbiota.	The title clearly reflects the observational nature of the study, investigating the effects of tobacco smoke exposure and breastfeeding duration on gut microbiota in children.	The title clearly reflects the observational nature of the study, investigating the effects of tobacco smoke exposure in prenatal and early postnatal life on infant gut microbiota and childhood overweight risk
Abstract	Does the abstract provide an informative and balanced summary of what was done and found?	The abstract provides a clear summary of objectives, methods, and key findings related to the microbiota types and their association with health outcomes	The abstract summarizes the objectives, methods (cohort study, 16S rRNA gene sequencing), and key findings about changes in microbial diversity due to maternal smoking.	The abstract summarizes the objectives, methods (cohort study, 16S rRNA sequencing), and key findings regarding maternal and environmental factors affecting gut microbiome diversity.	The abstract summarizes the objective, methodolog y (16S rRNA gene sequencing), and the key findings related to thirdhand smoke exposure and gut microbiome diversity.	The abstract provides a comprehensive summary of the objectives, methodology, and key findings, including maternal smoking effects on microbiome composition and obesity.	The abstract provides a concise summary of the study's objectives, methodology, and key findings, focusing on tobacco and mercury exposure and their associations with gut microbiota composition.	The abstract provides a comprehensiv e summary of the study's objectives, methodology (16S rRNA gene sequencing), and key findings on the relationship between tobacco exposure, breastfeeding duration, and gut microbiota diversity.	The abstract provides a comprehensiv e summary of the study's objectives, methodology (16S rRNA gene sequencing), and key findings on tobacco smoke exposure, gut microbiota changes, and childhood overweight risk.
**Introductio n**									
Background /Rationale	Is the scientific background and rationale for the	The introduction clearly explains the background	The introduction provides a clear rationale for the study, explaining the	The introduction provides a clear rationale for the study,	The introduction provides a solid rationale for	The introduction effectively outlines the connection between	The introduction effectively outlines the significance of	The introduction outlines the significance of understanding	The introduction outlines the significance of studying

Key STROBE Checklist Items	Questions	Gosalbes et al. [18]	Huotari et al. [20]	Levin et al. [23]	Northrup et al. [19]	Peng et al. [24]	Pérez-Castro et al. [17]	Xie et al. [22]	Tun et al. [21]
	study explained?	on meconium microbiota, its potential link to maternal factors, and its relevance to atopic diseases and other immune-related outcomes.	significance of studying the gut microbiome and how maternal smoking could impact it (Huotari, 2020).	emphasizing the importance of understanding environmental and sociocultural factors that influence gut microbiome development in early life.	studying the effects of thirdhand smoke exposure on the gut microbiome of infants, explaining the relevance of microbial diversity to infant health.	maternal smoking during pregnancy, gut microbiota, and childhood overweight/obesity risk, providing a strong rationale.	studying tobacco and mercury exposure during pregnancy and childhood, highlighting potential effects on the gut microbiota.	how environmental tobacco smoke exposure and breastfeeding duration affect gut microbiota, especially during early childhood, and their relevance to health outcomes.	tobacco smoke exposure and its impact on gut microbiota and childhood obesity, providing a strong rationale supported by previous research and meta-analyses.
Objectives	Are the specific objectives or hypotheses clearly stated?	The study objectives are clearly stated—to characterize the microbiota in term infants and assess its associations with maternal and infant health outcomes.	The objective is to assess whether maternal smoking during pregnancy affects the gut microbiome in the first-pass meconium of infants.	The objective is to assess the joint effects of pregnancy, sociocultural, and environmental factors on the gut microbiome structure and diversity in infants from a racially and socioeconomically diverse cohort.	The objective is clearly stated: to explore the associations between thirdhand smoke (THS) exposure and gut microbiome differences in NICU-admitted infants.	The objective is to assess the relationship between maternal smoking during pregnancy and gut microbiota's mediating effect on childhood obesity, along with potential microbial and metabolic mediators.	The study's objective is clearly stated: to assess how exposure to tobacco and mercury from pregnancy to childhood influences the gut microbiota of 7-year-old children.	The study's objectives are clearly stated: to explore the composition and development of gut microbiota in young children exposed to tobacco smoke and with varying breastfeeding durations over two years.	The study's objectives are clearly stated: to explore the associations between prenatal and postnatal tobacco smoke exposure, gut microbiota composition, and the risk of childhood overweight and obesity.
**Methods**									
Study Design	Is the key element of the study design (e.g., cohort, cross-sectional) presented early in the paper?	The article outlines that it is an observational study on microbiota in a birth cohort.	The study is observational, focusing on a cohort of 131 infants.	The article outlines that it is an observational cohort study of 298 children, with data collected through questionnaires and stool sample sequencing.	The article outlines an observational study of 43 mother-infant dyads admitted to the NICU, with stool samples analyzed using 16S rRNA sequencing.	The study is a large-scale observational cohort study involving 1,592 children from the Canadian Healthy Infant Longitudinal Development (CHILD) cohort.	The article details an observational study on 151 children from the INMA birth cohort, analyzing gut microbiota at 7 years of age using 16S rRNA sequencing.	The study employs a prospective cohort design, following 37 mother-child pairs in China over two years, collecting demographic information and fecal samples at different child age points (6,	The study employs a prospective cohort design, following 999 infants in three cities (Edmonton, Vancouver, Winnipeg) enrolled in the Canadian Healthy Infant Longitudinal Development

Key STROBE Checklist Items	Questions	Gosalbes et al. [18]	Huotari et al. [20]	Levin et al. [23]	Northrup et al. [19]	Peng et al. [24]	Pérez-Castro et al. [17]	Xie et al. [22]	Tun et al. [21]
								12, and 24 months).	(CHILD) cohort.
Setting	Is the setting, locations, and relevant dates (e.g., periods of recruitment and follow-up) clearly described?	Details about the study setting, including sample collection at La Fe Hospital in Valencia and the timeline of infant follow-ups, are clearly described.	The study took place at Central Finland Central Hospital, with meconium samples collected shortly after birth and analyzed at the University of Oulu.	The study was conducted in the Detroit metropolitan area, with stool samples collected from children at 1 and 6 months of age.	The study took place in a neonatal intensive care unit (NICU) in the Houston area, and the study timeline is clearly described.	The study setting is clearly defined, including data collection across different time points (prenatal to three years) with stool samples collected at 3 and 12 months.	The study was conducted in Sabadell, Catalonia, Spain, as part of the INMA Project, and the data collection timeline is well-documented.	The study was conducted at Zhongnan Hospital of Wuhan University in China, with fecal samples collected from mothers during late pregnancy and from their children at various time points.	The study was conducted across three Canadian cities, and the design includes prenatal and postnatal data collection, as well as fecal sample analysis at 3–4 months of age.
Participants	Are eligibility criteria and sources and methods of selection described?	Inclusion criteria (term newborns, healthy pregnancies) and sampling methods (meconium, fecal samples) are well-defined.	The study included 131 infants whose first-pass meconium was collected. Smoking data was reported by the mothers through questionnaires.	The study involved 298 children, with 130 neonates and 168 infants. Participants were recruited from a birth cohort based in Detroit (WHEALS cohort).	The inclusion criteria for the infants (NICU admission, thirdhand smoke exposure, consent from parents) and mothers are well-documented. However, only 43 dyads were included.	Inclusion criteria (e.g., singleton live births at ≥35 weeks of gestation, complete data on maternal smoking and microbiota) and exclusion criteria (e.g., in vitro fertilization, home births) are well-defined.	The inclusion criteria for the participants are clearly described, and 151 children from the initial 657 recruited mother–infant pairs were included in the final analysis.	The study included 37 mother-child pairs; inclusion criteria and exclusion criteria are clearly documented (e.g., non-smoking mothers, no antibiotic use during pregnancy).	The study included 999 infants. The exposure groups are clearly defined based on smoking exposure: no exposure, exposure during pregnancy only, exposure postnatally only, and exposure during both pregnancy and postnatally.
Variables	Are all variables (independent, dependent) clearly defined?	Variables like microbiota diversity, bacterial taxa abundance,	The key variables are microbial diversity and abundance, operational taxonomic units	Variables included maternal race-ethnicity, breastfeeding, mode of delivery,	Key variables include THS exposure, nicotine levels, urine cotinine,	Key variables include maternal smoking status, gut microbiota diversity, childhood BMI,	Key variables include exposure to tobacco (cotinine levels, second-hand smoke)	Key variables include environmental tobacco smoke exposure (measured through self-	Key variables include maternal smoking status, exposure to other

Key STROBE Checklist Items	Questions	Gosalbes et al. [18]	Huotari et al. [20]	Levin et al. [23]	Northrup et al. [19]	Peng et al. [24]	Pérez-Castro et al. [17]	Xie et al. [22]	Tun et al. [21]
		and health outcomes (eczema, respiratory problems) are clearly defined and analyzed.	(OTUs), and phyla such as Bacteroidetes and Firmicutes, which are	environmental tobacco smoke (ETS) exposure, and other sociodemographic factors.	and bacterial diversity in stool samples. Variables like gestational age, antibiotics, and breastmilk feeding are controlled for (Northrup, 2021).	and associated risk factors such as breastfeeding status and maternal pre-pregnancy BMI.	and mercury (cord blood, hair samples), as well as dietary and demographic determinants.	reported smoking exposure by family members) and breastfeeding duration, alongside microbiota diversity indices and bacterial composition.	household smokers, microbial diversity and richness, BMI z-scores, and covariates such as maternal BMI, breastfeeding, and antibiotics exposure.
Data Sources/Measurement	Are data sources and measurement methods clearly described, including their validity?	Molecular methods like 16S rDNA sequencing are detailed for data collection, and statistical analysis is described in terms of logistic regression and diversity indices.	Data on the gut microbiome were obtained via 16S rRNA gene sequencing. Diversity indices (Shannon, Simpson) were calculated and compared between infants of smoking and non-smoking mothers.	Gut microbiota were profiled using 16S rRNA sequencing. Statistical analyses (PERMANOVA, UniFrac distances) were used to evaluate the impact of various factors on microbial composition.	Data collection methods, including the collection of stool, urine cotinine, and surface nicotine, are clearly described, as well as the DNA sequencing techniques used to analyze the microbiome.	Data collection methods are described in detail, including questionnaires, microbiome analysis via 16S rRNA sequencing, and metabolomics to analyze gut metabolites.	Data sources such as cord blood for mercury and urine for cotinine were used to measure exposure, while microbiome composition was determined through 16S rRNA sequencing.	The study used 16S rRNA gene sequencing to analyze gut microbiota and fecal samples at different time points. Demographic data and tobacco smoke exposure frequency were collected through self-reported questionnaires.	Tobacco exposure data were collected through maternal and household questionnaires, and gut microbiota were analyzed using 16S rRNA sequencing from infant fecal samples at 3–4 months. Weight and height were measured to calculate BMI z-scores.
Bias	Are the potential sources of bias addressed?	Potential biases, such as contamination from external sources during sample collection, are addressed by separating	Potential biases include the small sample size and the limited number of smoking mothers (n=5). Environmental tobacco exposure was not accounted for.	Biases such as self-reporting and limitations due to the size of the cohort (limited number of smoking mothers) are acknowledged.	The potential biases related to self-reporting and the limited sample size are discussed. However, the study acknowledg	The study addresses potential biases, including self-reported smoking data and possible confounding factors like breastfeeding status. Oversampling from smoking households is	The study acknowledges potential biases, including self-reported smoking exposure and the challenge of adjusting for all environmental factors. A sensitivity	Biases are acknowledged, such as potential recall bias in self-reported tobacco exposure and the relatively small sample size. A sensitivity analysis was performed to	The study acknowledges potential biases, including self-reported smoking exposure and the challenge of adjusting for maternal overweight as a confounder.

Key STROBE Checklist Items	Questions	Gosalbes et al. [18]	Huotari et al. [20]	Levin et al. [23]	Northrup et al. [19]	Peng et al. [24]	Pérez-Castro et al. [17]	Xie et al. [22]	Tun et al. [21]
		internal and external portions of meconium.			es oversampling from smoking households to reduce bias.	used to minimize bias.	analysis was performed to control for genetic factors.	control for confounding factors such as pet ownership and alcohol consumption.	
Study Size	Is the rationale for the study size explained?	The rationale for the study size is not explicitly explained in the article.	The study size is relatively small, and the authors acknowledge that larger studies are needed to confirm the results.	The study size of 298 children is considered sufficient for the analysis, though larger studies are recommended to confirm these findings.	The rationale for the study size is not fully explained, though the authors recognize that this is an exploratory study with 43 dyads, and larger studies are needed.	The rationale for the study size is implied by the large sample of 1,592 children, which is considered sufficient for statistical power.	The study size (151 children) is justified as exploratory, with a focus on examining multiple exposures. Larger follow-up studies are recommended.	The study size (37 pairs) is relatively small, and the authors suggest larger studies are needed for further validation of the findings.	The sample size of 999 infants is sufficient for robust statistical analysis, and larger follow-up studies are suggested.
Quantitative Variables	Are methods for how quantitative variables were handled explained?	Methods for how quantitative variables were handled are explained in terms of diversity indices and statistical models.	Quantitative methods such as the Shannon and Simpson diversity indices and the Chao1 index were used to measure microbial diversity.	Quantitative variables like diversity indices (Shannon, Simpson) and UniFrac distances were used to measure differences in gut microbiome composition.	Quantitative variables include alpha and beta diversity indices (Shannon, Simpson), operational taxonomic units (OTUs), and the relative abundance of specific bacterial taxa.	The study used quantitative measures like alpha and beta diversity indices (Chao1, Faith's PD) and BMI z-scores to assess microbial diversity and obesity risk.	Quantitative variables include microbiota diversity indices, bacterial abundance, and pollutant exposure levels (e.g., cotinine, mercury	Quantitative measures include microbiota diversity indices (Shannon, Simpson) and bacterial abundance across genera. Variables such as breastfeeding duration and smoking exposure frequency were analyzed using regression models	Quantitative measures include microbiota diversity indices (species richness of Firmicutes, Ruminococcaceae abundance), BMI z-scores, and the odds ratios for overweight and obesity.
Statistical Methods	Are the statistical methods, including subgroup	Statistical methods are described, including logistic	Statistical analyses were conducted using SPSS software. The Mann-	Statistical methods included PERMANOVA and zero-	The article describes the use of negative binomial	Generalized linear models, mediation analysis, and correlation tests	Statistical methods include PERMANOVA for diversity	Statistical analyses included Welch's t-tests to compare	Statistical analyses included logistic regression for

Key STROBE Checklist Items	Questions	Gosalbes et al. [18]	Huotari et al. [20]	Levin et al. [23]	Northrup et al. [19]	Peng et al. [24]	Pérez-Castro et al. [17]	Xie et al. [22]	Tun et al. [21]
	analysis, described in detail?	regression and Mann–Whitney tests for comparing clinical associations with microbiota.	Whitney U-test was used to compare microbial abundances between groups.	inflated negative binomial regression for analyzing the effects of factors on microbiome composition.	regression, Mann-Whitney U-tests, and Bayesian analyses to model the associations between THS exposure and microbiome diversity.	(Pearson's, Spearman's) are used to assess the relationship between maternal smoking and obesity.	measures and MaAsLin2 for genus-level associations, adjusted for covariates like maternal education and BMI.	alpha diversity between groups, PERMANOVA for beta diversity differences, and multivariate regression models adjusted for confounders.	overweight/obesity risk, adjusted odds ratios (OR), and mediation analysis to explore gut microbiota's role in the association between smoking exposure and obesity.
**Results**									
Participants	Are numbers of participants at each stage (e.g., recruitment, follow-up, analysis) reported?	The flow of participants is reported clearly—20 term newborns and their mothers were included in the study.	The study included 131 infants, with 5 mothers reporting smoking during pregnancy.	The study clearly describes the inclusion of 130 neonates and 168 infants from the WHEALS cohort, providing detailed demographic data.	The study included 43 mother-infant dyads, and the article reports their characteristics in terms of gestational age, antibiotic use, and other relevant factors.	The study reports characteristics of the participants, including BMI categories (normal, overweight, obese) and their association with maternal smoking.	The article reports detailed participant characteristics, including demographic data, exposure levels, and potential confounders like breastfeeding and diet.	The demographic characteristics of the 37 participants, including breastfeeding duration and tobacco smoke exposure, are well-documented.	The study reports detailed participant characteristics, including the number of infants exposed to tobacco smoke, their BMI z-scores, and gut microbiota diversity at 3–4 months.
Descriptive Data	Are demographic and clinical characteristics of study participants presented?	The demographic characteristics of mothers and infants (e.g., BMI, delivery method) are well-documented.	Data on the number of OTUs, as well as Shannon and Simpson diversity indices, were provided, showing a reduction in diversity in infants exposed to maternal smoking.	Descriptive statistics on participants' characteristics (e.g., breastfeeding status, race-ethnicity) are provided.	Descriptive statistics of the infants (e.g., antibiotic use, breastfeeding status, gestational age) are provided. The authors report the microbiome diversity differences.	Descriptive statistics are provided for participant characteristics (e.g., breastfeeding status, maternal pre-pregnancy BMI) and the microbiome diversity data.	Descriptive statistics on the frequency of tobacco and mercury exposure, as well as gut microbiota composition at the genus level, are provided.	Descriptive data include the relative abundance of key bacterial genera, such as Bifidobacterium, Veillonella, and Lactobacillus, at 6, 12, and 24 months.	Descriptive data include the relative abundance of Firmicutes and Ruminococcaceae in infants, and the prevalence of overweight/obesity at ages 1 and 3.

Key STROBE Checklist Items	Questions	Gosalbes et al. [18]	Huotari et al. [20]	Levin et al. [23]	Northrup et al. [19]	Peng et al. [24]	Pérez-Castro et al. [17]	Xie et al. [22]	Tun et al. [21]
Outcome Data	Are main outcomes and measures clearly reported?	The main outcomes, including microbiota types and their association with maternal eczema and respiratory issues in infants, are clearly reported with appropriate confidence intervals and statistical values.	The main outcome was a decrease in microbial diversity and an increase in Bacteroidetes and Proteobacteria phyla in infants exposed to maternal smoking.	The primary outcomes include changes in gut microbiota composition, including the relative abundance of specific bacterial taxa (e.g., Bifidobacteriaceae, Lachnospiraceae).	The main outcomes include lower alpha diversity in infants from smoking households and associations between THS-related exposure (cotinine, surface nicotine) and specific bacterial genera.	The primary outcomes are BMI z-scores and microbial diversity indices, with specific microbial taxa (e.g., Firmicutes, Lachnospiraceae) associated with smoking exposure and obesity.	The main outcomes include the impact of prenatal tobacco exposure on gut microbiota diversity and changes in specific genera like Akkermansia and Dorea.	The main outcomes include higher gut microbiota diversity in non-smoke-exposed children and increased Lactobacillus abundance with prolonged breastfeeding.	The main outcomes show a significant association between postnatal tobacco smoke exposure and increased overweight/obesity risk, with increased gut microbiota diversity.
Main Results	Are unadjusted estimates and, if applicable, adjusted estimates with confidence intervals provided?	Unadjusted and adjusted estimates are available in several places. For instance, odds ratios (OR) with 95% confidence intervals (CI) were used for measuring associations, particularly for variables like maternal age, gestational age, and smoking status during pregnancy. Additionally, odds ratios and	The article reports the mean values of diversity indices and relative abundance levels for different microbial phyla, categorized by exposure to maternal smoking.	The study does include univariate and multivariate models (PERMANOVA) where factors like maternal smoking and ETS are associated with variations in gut microbiome composition in neonates and infants. These models focus on compositional changes (e.g., variations in specific taxa) rather than unadjusted or adjusted estimates like	Bayesian statistical inference is used in this study, and probabilities are provided instead of traditional confidence intervals. The study discusses posterior probabilities (PP) as indicators of evidence strength. For certain bacterial taxa, models include adjustments for covariates like gestational age and antibiotics	In the generalized linear models assessing BMI z-scores, children exposed to maternal smoking had higher BMI z-scores at age 1 year (unadjusted: β = 0.27, 95% CI 0.07–0.47) and at age 3 years (adjusted: β = 0.28, 95% CI 0.06–0.49).<br>The odds of becoming overweight or obese by age 3 years were significantly higher for children exposed to maternal smoking (adjusted: OR	The study contains both unadjusted and adjusted estimates with confidence intervals (CIs) for various environmental and pollutant exposure factors, specifically tobacco and mercury exposure.	both unadjusted and adjusted estimates are provided. Unadjusted models assess the impact of factors such as sibling presence and tobacco exposure during pregnancy, while adjusted models account for various determinants and pollutant exposures. These adjustments include consideration of factors like gestational age, maternal education, and environmental	Both unadjusted and adjusted estimates with confidence intervals are provided. Unadjusted odds ratios (OR) for overweight/obesity are reported, such as OR 2.10 (95% CI: 1.13–3.91) for postnatal tobacco smoke exposure and OR 3.32 (95% CI: 1.42–7.75) for combined prenatal and postnatal exposure at age 1 year. Adjusted estimates are also provided, accounting for

Key STROBE Checklist Items	Questions	Gosalbes et al. [18]	Huotari et al. [20]	Levin et al. [23]	Northrup et al. [19]	Peng et al. [24]	Pérez-Castro et al. [17]	Xie et al. [22]	Tun et al. [21]
		confidence intervals for microbiota types, labor initiation, delivery method, and breastfeeding are mentioned		odds ratios or hazard ratios.	use, with Bayesian thresholds replacing confidence intervals.	1.78, 95% CI 1.11–2.86).		exposures to better understand their influence on children's gut microbiota composition and diversity	factors like maternal prenatal smoking, which attenuated the associations in some cases. Confidence intervals accompany all the estimates, ensuring statistical significance is appropriately reported. 4o
**Discussion**									
Key Results	Are key results summarized in relation to the study's objectives?	The results are summarized in the context of the study's objectives, with interpretations of the relationship between microbiota composition and health outcomes.	The results indicate that maternal smoking is associated with lower microbial diversity and changes in phylum-level composition in the infant gut microbiome.	The study highlights the importance of sociocultural and environmental factors, such as maternal race-ethnicity and ETS exposure, on gut microbiota composition in early life.	The key results are summarized, showing that higher THS exposure is associated with lower gut microbiome diversity and a lower abundance of Bifidobacterium and other taxa.	The results demonstrate the association between maternal smoking and increased gut microbiota diversity, especially Firmicutes, contributing to a higher obesity risk in children.	The key results suggest that prenatal tobacco exposure is associated with long-term effects on gut microbiota composition, while mercury exposure was not significantly associated with microbiota changes.	The study found that prolonged breastfeeding was positively associated with beneficial bacteria (Lactobacillus), while tobacco smoke exposure negatively affected microbiota diversity	The study found that household tobacco smoke exposure postnatally and during both pregnancy and postnatally increases the risk of childhood overweight and obesity, potentially mediated by gut microbiota changes.
Limitations	Are study limitations, potential sources of bias, and imprecision discussed?	The authors acknowledge the small sample size and the need for further	The authors acknowledge the small sample size and the lack of data on environmental smoking	Limitations include the small sample size and the reliance on self-reported data for	The authors acknowledge the small sample size, potential biases in self-	The authors acknowledge the lack of maternal gut microbiota data and epigenetic analyses as	The authors acknowledge limitations, including the small sample size, lack of species-level	The study acknowledges limitations, including the small sample size, reliance on self-	The authors acknowledge limitations such as the potential for residual confounding

Key STROBE Checklist Items	Questions	Gosalbes et al. [18]	Huotari et al. [20]	Levin et al. [23]	Northrup et al. [19]	Peng et al. [24]	Pérez-Castro et al. [17]	Xie et al. [22]	Tun et al. [21]
		studies to confirm the associations.	exposure as key limitations.	certain factors (e.g., breastfeeding, smoking status).	reporting, and the need for further studies with larger cohorts to validate these findings.	limitations, along with potential residual confounding from unmeasured variables.	resolution in microbiota analysis, and possible confounding factors.	reported tobacco exposure data, and lack of quantitative measures of tobacco exposure.	from unmeasured environmental factors, as well as the reliance on self-reported smoking data.
Interpretation	Are results interpreted in the context of existing evidence?	The results are compared with previous research on microbiota and its role in immune modulation.	The findings align with existing research on the effects of smoking on adult gut microbiomes, but further research is needed to explore the prenatal impact.	The findings are consistent with existing literature on the impact of maternal and environmental factors on gut microbiome development, but further research is recommended.	The findings are consistent with previous studies on the effects of smoke exposure on gut microbiota, but the authors call for more comprehensive research on this topic.	The findings align with previous studies on smoking and obesity, providing novel insights into the microbiota-mediated mechanisms linking maternal smoking to childhood obesity.	The findings are consistent with prior studies on tobacco's impact on microbiota but highlight the need for more detailed analyses of mercury's effects.	The findings align with previous research showing that environmental factors such as tobacco smoke and breastfeeding influence gut microbiota composition, but further studies are needed to confirm these results.	The findings align with existing research linking prenatal and postnatal tobacco smoke exposure to childhood overweight, and further emphasize the role of gut microbiota as a mediator in this relationship.
Generalizability	Are the generalizability (external validity) of the study results discussed?	The authors discuss the limitations regarding the generalizability of their findings, given the small cohort and the potential for variability across populations.	The authors discuss the limited generalizability of the study due to the small sample size.	The study focuses on a racially and socioeconomically diverse cohort, but the authors acknowledge that generalizability may be limited to similar populations.	The study recognizes the limited generalizability due to the small sample size and the specific population of NICU-admitted infants.	The large cohort size enhances the generalizability of the findings, though the authors note that further validation in other populations is needed.	The study's results are specific to a Mediterranean cohort, and the authors note that generalizability may be limited to similar population.	The study's findings are specific to a Chinese cohort, and the authors note that generalizability may be limited to similar populations and larger studies are needed	The study's findings are generalizable to other populations with similar household smoking exposure, but further research is recommended to validate the findings in more diverse cohorts.
**Other Information**									
Funding	Is the source of funding and the role of the	The funding sources and acknowledg	The study was conducted as part of the	Funding sources and acknowledgm	The article provides details on	The study provides detailed	The study provides a clear	The study reports funding from	The study provides clear statements

Key STROBE Checklist Items	Questions	Gosalbes et al. [18]	Huotari et al. [20]	Levin et al. [23]	Northrup et al. [19]	Peng et al. [24]	Pérez-Castro et al. [17]	Xie et al. [22]	Tun et al. [21]
	funders in the research clearly stated?	ments are clearly provided, with no reported conflicts of interest.	University of Oulu's research program, with funding acknowledged but no conflicts of interest reported.	ents are clearly provided, with no conflicts of interest reported.	the funding sources (National Institutes of Health) and notes that the authors have no competing financial interests.	information on funding sources, including grants from the Canadian Institutes of Health Research and the Research Grants Council of Hong Kong.	statement of funding sources, including European Union's Horizon 2020 research programs.	the National Natural Science Foundation of China, with no conflicts of interest disclosed	about funding sources and declares no conflicts of interest.

16S rRNA - 16S Ribosomal RNA; **BMI** - Body Mass Index; **CI** - Confidence Interval; **CHILD** - Canadian Healthy Infant Longitudinal Development; **ETS** - Environmental Tobacco Smoke; **NICU** - Neonatal Intensive Care Unit; **OR** - Odds Ratio; **OTU** - Operational Taxonomic Unit; **PP** - Posterior Probability; rRNA - Ribosomal RNA; **THS** - Thirdhand Smoke; **WHEALS** - Wayne County Health, Environment, Allergy, and Asthma Longitudinal Study
